# IEEE P2668 Compatible Evaluation Strategy for Smart Battery Management Systems

**DOI:** 10.3390/s22166057

**Published:** 2022-08-13

**Authors:** Hao Wang, Kim Fung Tsang, Chung Kit Wu, Yang Wei, Yucheng Liu, Chun Sing Lai

**Affiliations:** 1 Department of Electrical Engineering, City University of Hong Kong, Hong Kong 999077, China; 2 Brunel Interdisciplinary Power Systems Research Centre, Department of Electronic and Electrical Engineering, Brunel University London, London UB8 3PH, UK

**Keywords:** IEEE P2668, Internet-of-Things, smart battery management

## Abstract

In smart cities and smart industry, a Battery Management System (BMS) focuses on the intelligent supervision of the status (e.g., state of charge, temperature) of batteries (e.g., lithium battery, lead battery). Internet of Things (IoT) integration enhances the system’s intelligence and convenience, making it a Smart BMS (SBMS). However, this also raises concerns regarding evaluating the SBMS in the wireless context in which these systems are installed. Considering the battery application, in particular, the SBMS will depend on several wireless communication characteristics, such as mobility, latency, fading, etc., necessitating a tailored evaluation strategy. This study proposes an IEEE P2668-Compatible SBMS Evaluation Strategy (SBMS-ES) to overcome this issue. The SBMS-ES is based on the IEEE P2668 worldwide standard, which aims to assess IoT solutions’ maturity. It evaluates the characteristics of the wireless environment for SBMS while considering battery factors. The SBMS-ES scores the candidates under numerous scenarios with various characteristics. A final score between 0 and 5 is given to indicate the performance of the SBMS regarding the application demands. The disadvantages of the SBMS solution and the most desired candidate can be found with the evaluated score. SBMS-ES provides guidance to avoid potential risks and mitigates the issues posed by an inadequate or unsatisfactory SBMS solution. A case study is depicted for illustration.

## 1. Introduction

Battery sales are growing in the global market. As predicted by Grand View Research, the global battery market size achieved USD 108.4 billion in 2019, and it is expected that growth with a Compound Annual Growth Rate (CAGR) of 14.1% from 2020 to 2027 will occur [1]. The growth of the battery market, including lithium and lead-acid batteries, is mainly attributed to the demand for automotive and renewable energy applications [2,3]. Besides, batteries are employed in other areas, such as solar power plant energy storage, data centers, offshore drilling platforms, north and south poles, and airplane and vehicle cranking [4].

The usage of batteries requires extra attention, particularly in the critical applications. Otherwise, an inappropriate installation or use may cause additional costs or even accidents such as fires or explosions. The main risk of the battery usage is from the operating temperature, influenced by the internal chemical processes of the battery [5]. The reasons are twofold: on the one hand, the life of batteries decreases substantially when the temperature rises. As a result, the battery’s maintenance expenses will increase. On the other hand, the effects can be devastating when batteries are exposed to severe temperatures. The expansion of frozen electrolytes at low temperatures and State-of-Charge (SOC) can cause battery ruptures. In addition, uncontrolled reactions, thermal runaway, may occur due to a high operating temperature. A thermal runaway is a self-heating process that leads the battery to shut down or explode [6,7]. Due to the positive net heat energy in processes, the exothermic reaction in batteries is self-sustaining. Hence, the safe employment of batteries raises many concerns considering these mentioned risk features. Furthermore, extreme caution should be particularly paid when batteries are deployed in critical applications, to prevent severe consequences.

Hence, monitoring battery status during the operation is essential to prevent risks [8]. Smart Battery Management Systems (SBMSs) are proposed to complete such tasks by implementing the supervision of various critical features, such as operating temperature, State of Health (SOH), SOC, etc. Traditional BMSs employ Controller Area Network (CAN)-bus and I2C/SPI communication protocols. However, traditional BMSs are believed to have unreliability, high cost, and complexity as negatives, which have resulted in the emergence of new types of BMS [9]. Compared to conventional BMS, the SBMS applies wireless communication methods to report the monitoring results, providing improved reliability, lower cost, and sensor deployment feasibility [9]. With wireless communication technologies employed, the SBMSs is recognized as one of the many Internet-of-Things (IoT)-based smart applications. The IoT refers to the connection of physical items (“things”) equipped with various elements and technologies, including sensors, software, etc., to exchange data with other devices and systems through the internet [10]. These physical objects can share and gather data with minimum human interaction, using state-of-art technologies, such as big data analytics, cloud computing, and mobile communications.

Various categories of SBMS, including experimental or model-based methods, have been presented to contribute to battery status such as temperature, etc. However, the SBMS also brings new challenges. A failure may occur if the wireless communication techniques are inappropriately applied. For example, wireless communication techniques with high latency are unsuitable for time-critical SBMSs. However, a general SBMS evaluation strategy is lacking to address this challenge. As a result, developers are not able to evaluate the performance of their SBMS, and it is hard to determine the best configuration for their applications.

To solve this problem, the SBMS evaluation strategy (SBMS-ES) is proposed in this paper. The SBMS-ES is a comprehensive evaluation strategy which considers the impact of the IoT on the SBMS. Integrating the IEEE P2668 global standard makes the SBMS-ES a general strategy that could be widely applied. The SBMS-ES identifies the essential features of the SBMS and designs a scoring guideline for each of them. A weighted average value is calculated as the final score, based on the evaluated sub-scores of attributes, to indicate the overall performance of the SBMS. The weighting is obtained through implementing an Analytic Hierarchy Process (AHP), a broadly utilized decision-making procedure. The final score is applied to rank the candidate SBMS solutions, to find the most desired one regarding the scenario demands.

The contributions are as follows:

1. The SBMS-ES is presented, the first of its kind, to evaluate the performance of the SBMS. With SBMS-ES, SBMS designers can determine the optimum configuration referring to the evaluated score.
2. Critical features for SBMS evaluation are identified in SBMS-ES. A general scoring scheme is designed, based on the identified features.
3. A case study is illustrated, to describe the usage of the SBMS-ES.

The structure of the paper is as follows: The relevant works associated with SBMS are addressed in Section 2. Then, Section 3 offers specifics about SBMS-ES. Section 4 depicts a case study, to describe the implementation of SBMS-ES. Section 5 summarizes and concludes.

## 2. Related Works

Cloud computing and IoT have been widely utilized by researchers, based on traditional BMS, to design SBMS solutions. Kim et al. [11] introduced IoT-enabled battery conditional monitoring and fault diagnosis for Li-ion large-scale applications. Moreover, a digital battery twin was developed, by combining battery monitoring and data-driven modeling approaches. In [12], an SOC estimate method for lithium-ion and lead-acid batteries was based on an adaptive extended H-infinity filter. In addition, a state-of-health estimate system with particle swarm optimization was designed to monitor the battery’s capacity and power degradation as it ages. With cloud computing and IoT, a digital twin was built to implement monitoring simultaneously. Xinrong et al. [13] proposed a Wireless Smart Battery Management System (WSBMS) to manage battery cells in electric vehicles (EVs). The developed system aimed to improve performance in fault tolerance and scalability. A balancing algorithm was presented to balance battery cells with various features, such as numbers. Friansa et al. [14] suggested an IoT-based battery monitoring system for microgrid batteries. A human–machine interface was designed using an ExtJS/HTML5 framework to store information, which can be accessed on a desktop. Tetsu et al. [15] developed a cloud-connected battery management system that monitors shared batteries’ status. The designed system continually connects to the batteries, managing their SOC and monitoring changes in their attributes via a location data cloud. It supports e-mobility and can be applied to Electric Vehicles (EV). The authors of [16] presented a smart battery management system to prolong battery life. Authors of [17] proposed a control strategy to minimize the side reaction-induced capacity loss, by changing the cell series-parallel configuration dynamically inside the battery pack.

In addition to the aforementioned SBMS, researchers also have proposed works aiming to study the performance of the wireless communication protocol in the BMS. Alonso et al. [18] researched wireless channel parameters and data rates in a BMS. The main work was to estimate the transmission capacities of different antenna types in various frequency bands. The study also concentrated on Planar Inverted-F- Antenna and CAN-bus communication. Kumtachi et al. [19] improved the reliability of a multi-hop wireless communication protocol for BMS electric vehicles. Specifically, the approach achieves successful communication within 20 ms for over 99% of packets by overhearing those incoming packets without optimal routes.

The mentioned works have made remarkable contributions to the study of SBMS. However, these works solely focused on battery or communication performance monitoring in BMS. The field of SBMS lacks a systematic strategy for evaluating the overall performance of solutions. As a result, designers cannot decide the best configuration for their SBMS. A comprehensive evaluation of SBMS is necessary.

## 3. P2668 Interoperable Standardized Management Framework

### 3.1. IEEE P2668 Global Standard

The IEEE P2668 standard defines methods and criteria for evaluating the performance of IoT objects, the evaluation outcome of which is expressed as a quantitative indicator, namely the IDex [20]. The IDex categorizes the maturity of (IoT) objects into five levels, ranging from one (the lowest maturity) to five (the most excellent maturity) [21]. The final IDex value is expected to satisfy the requirement of IoT stakeholders for a clear indication. IDex can also be used to forecast performance changes under various operating circumstances and to present recommendations for increasing the performance of IoT objects. The main objective of IDex is to evaluate the performance of IoT solutions and provide advice on corresponding improvements.

### 3.2. Overview of SBMS-ES

The SBMS can be evaluated by IDex since it utilizes IoT technology. The specialized scheme for the employment of IDex in the SBMS is called SBMS-ES (evaluation scheme). This section introduces the general construction of SBMS-ES, step by step. The quantitative score of each SBMS solution can be obtained to its comprehensive performance, applying SBMS-ES. By comparing the final scores of SBMS with various configurations (e.g., different communication protocols), the best solution among the candidates can be decided.

**Figure 1:**
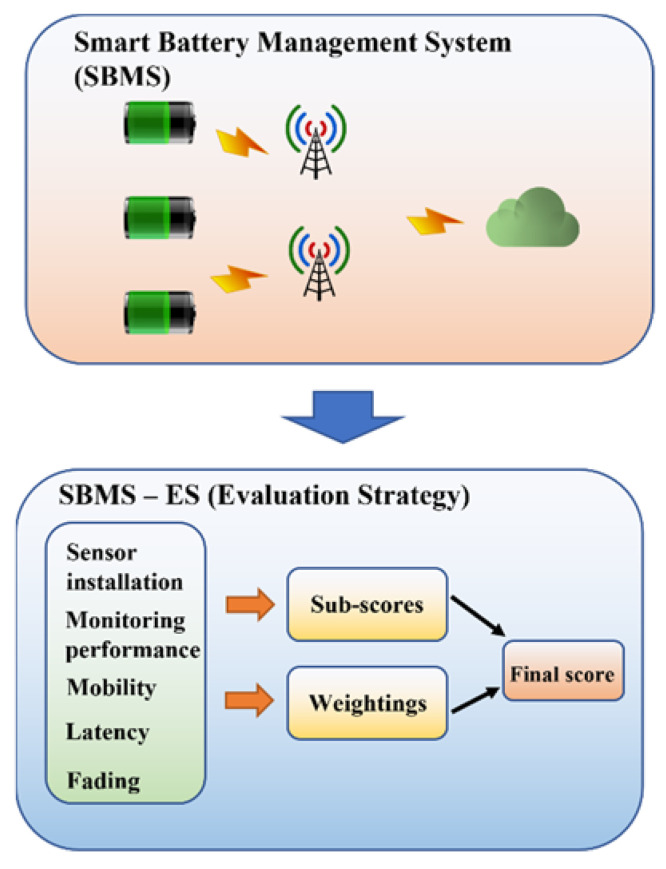
Flowchart of SMBS-ES.

A flowchart of SBMS-ES is illustrated in Figure 1. It is divided into three subsections, i.e., the attributes evaluation, the weighting allocation, and the final score calculation. The attribute evaluation introduces the identified essential attributes in SBMS-ES and the justifications. Furthermore, the evaluation principles of the attributes are illustrated. The weighting allocation describes how the weighting for each attribute is determined. The final score calculation depicts the way to calculate the final score and select the most desired SBMS solution. The details of these subsections are specified in the following Section 3.3, Section 3.4 and Section 3.5.

### 3.3. Evaluation Attributes in SBMS

Five key attributes are typically identified in SBMS-ES for evaluation, i.e., sensor installation, monitoring performance, mobility, latency, and fading. The attribute descriptions and sub-scores evaluation principles for each attribute are given in this section.

#### 3.3.1. Senor Installation

As mentioned, the SBMS implements battery status monitoring based on the relative sensors’ feature measurements. To be specific, a straightforward method directly measures the battery status of concern. On the opposite, the indirect method measures the other features to implement data modeling, i.e., estimating the status of concern based on the measured features. Both methods will need sensors for the measurement. Hence, the installation of sensors is part of the SBMS evaluation.

Sensor installation is evaluated in two aspects, i.e., the location of sensors, and the number of sensors. As discussed previously, installing a sensor inside a battery will change the original structure, which entails risks and extra costs. On the contrary, the influence is limited if the sensors are installed outside the battery, e.g., fixed on the battery surface. Hence, it is encouraged to install sensors outside.

Moreover, the number of sensors utilized to obtain the concentrated battery status will be considered when evaluating SBMS. The monitoring scheme that needs more measurements will require more sensors to be deployed, which will bring an increase of installation costs. Moreover, a larger packet size is requested by such schemes for data transmission. The monitoring scheme with fewer sensors is more recommended for the SBMS when the monitoring performance is consistent.

#### 3.3.2. Monitoring Performance

The monitoring performance represents the estimation accuracy of the battery status, which can be measured by the Mean Absolute Error (MAE) [22]. The value of the MAE of the SBMS needs to be as low as possible to improve its evaluation score of this aspect.

#### 3.3.3. Mobility

The mobility of a communication network is the technology that enables nodes to make communications with a moving status. A moving node that employs a communication technique without the function of mobility will suffer from poor communication quality.

The SBMS application scenario can be stationary or mobile [12]. Considering this, the mobility of the applied IoT technology in SBMS needs to be considered. If the battery (such as a lead-acid battery) is utilized in a moving vehicle, the capacity for mobility of the network is essential. Otherwise, mobility is not important in the SBMS if the batteries are fixed in the application scenario.

#### 3.3.4. Latency

Latency is the period between the point where the transmission signal is generated and the point where the same signal is received. In time-critical applications, the latency is decisive. Otherwise, the SBMS may fail.

#### 3.3.5. Fading

Fading indicates the loss of signal strength between the transmitter and the receiver. It is an essential parameter for calculating the received signal strength. In brief, the received signal strength can be calculated using the Equivalent Isotropically Radiated Power (EIRP) minus loss caused by fading. The transmission may fail if the received signal strength is lower than the sensitivity of the receiver. Fading is generated when the signal encounters, and becomes reflected, diffracted, and distributed by, obstacles along its transmission path. It is necessary to simulate fading using a propagation model before network installation. Low-quality communication will be caused if the demands of fading are not satisfactory.

There are two distinct forms of fading [23]. Small-scale fading is the short-term variation in the signal envelope induced by a local multipath. This is noticed at distances of around half a wavelength. Large-scale fading is the second form of fading.

Rician fading is a regular small fading, which is illustrated as an example. The probability density function (PDF, i.e., the density of a continuous random variable) of Rician fading is given in Equation (1), as follows [24]:

(1) $$f_{Rician}\left(x\right)=2x\frac{K+1}{\Omega_{R}}\exp\left(-K-x\frac{\left(K+1\right)x^{2}}{\Omega_{R}}\right)I_{0}\left(2x\sqrt{\frac{K\left(K+1\right)}{\Omega_{R}}}\right)$$

where *K* denotes the ratio of the power of the Light-of-Sight (LoS) signal component to the power of the other NLoS signal components, $\Omega_{R}$ represents the average power of the received signal, and *I*_0_ is the 0th order modified Bessel function of the first kind.

There are multiple kinds of propagation models developed for various scenarios (e.g., indoor, outdoor) and terrains (e.g., urban, rural), etc. The proper models should be implemented according to the application demands. Not all propagation models are detailed in this article, considering the page limit.

#### 3.3.6. Scoring

The scoring principles for attributes of SBMS-ES are shown in Table 1. The user of the SBMS-ES needs to check the performance of the candidate solution from the identified five aspects. As illustrated, there are five qualified levels for the attributes (highest 5). Regarding the sensor installation, the installation positions and the number of sensors are evaluated. If the sensors are installed outside the battery, and the no. of sensors is small, a score of 5 is evaluated. The monitoring performance is indicated by the MAE, which illustrates the difference between the estimated and real values. Similarly, a score of 5 will be given if the MAE of the predicted value (of temperature, SOC, etc.) is lower than 0.5%. Mobility checks whether the scenario requires mobility and whether the employed protocol supports it. The highest assessment score is obtained if both the mobile and stationary application scenarios can be satisfied. Moreover, the latency demanded by the application and achieved by the SBMS solution is examined. A score of 5 is provided if the application requires low latency and this is satisfied. Finally, the fading is modeled to check whether the signal can be transmitted successfully. The highest score can be given when the most strict fading requirement is achieved.

### 3.4. Weighting Allocation in SBMS-ES

Five essential attributes are identified and discussed in the above sections. After evaluating these attributes, a final grade is necessary, as a comprehensive estimation of all aspects. A reasonable weighting allocation is required to calculate the final grade and to obtain the final score.

The weighting allocation utilizes a decision-making method, namely the analytic hierarchy process (AHP), which was developed by Saaty et al. [25] to make decisions in multi-criteria decision-making (MCDM) problems. In MCDM problems, multiple criteria and alternatives exist, and the decision-maker needs to determine the best alternative based on the importance of the criteria. A rough description of the AHP steps is as follows.

At first, the AHP users need to compare the relative degree of importance between every two attributes. A score with a nine-point scale is given as the comparison result. The correspondence between the score and the degree of relative importance is shown in Table 2 [25]. If reversed, the reciprocal of the score will be considered as the result of the comparison. For instance, if attribute A is extremely more important than attribute B, a score of nine is given as the comparison result for A to B. Conversely, a score of 1/9 is given for B to A.

All identified attributes need to be compared pairwise. Assume that there are *N* attributes, then the paired comparison needs to be performed (N+1)N/2 times. A pairwise comparison matrix is obtained, to illustrate the comparison results, which is indicated by Equation (2).

(2) $$C=\left[\begin{array}{ccccc} 1 & c_{12} & c_{13} & \cdots & c_{1N}\\ c_{21} & 1 & c_{23} & \cdots & c_{2N}\\ c_{31} & c_{32} & 1 & \cdots & c_{3N}\\ \vdots & \vdots & \vdots & \ddots & \vdots \\ c_{N1} & c_{N2} & c_{N3} & \cdots & 1 \end{array}\right]$$

where *c_ij_* denotes the relative importance for attribute *i* to attribute *j*.

Then, the weighting vector *w* that denotes the weighting allocation is the normalized eigenvector corresponding to the maximal eigenvalue λmax:

(3) A×I=λmax×I 

(4) $$w=\frac{I}{\left\|I\right\|}$$

where *I* is the eigenvector corresponding to λmax.

Before applying the obtained weighting vector, it is necessary to validate whether the pairwise matrix is consistent. For instance, assume that attribute A is extremely important compared to attribute B, but of very strong importance compared to attribute C. It will be not reasonable if attribute B is more important than attribute C. Such a condition can be prevented if the comparison consistency checking is passed.

To examine the comparison consistency, the Consistency Ratio (*CR*) is calculated using the Consistency Index (*CI*) and Random Index (*RI*).

(5) $$CR=\frac{CI}{RI}=\frac{\left(\lambda_{max}-N\right)/\left(N-1\right)}{RI}$$

where *RI* values are defined in Table 3 [25].

The weighting vector *w* that passes the consistency checking will be utilized to assign weighting for each attribute and calculate the final score in next section.

### 3.5. Final Score Calculation in SBMS-ES

With the evaluated sub-scores and weightings for all attributes, the final score can be calculated using the formula below.

(6) $$S_{final}=\overset{5}{\underset{I}{\sum }}S_{sub,i}\times w_{i}$$

where $S_{sub,i}$ and $w_{i}$ represent the sub-score and weighting for the attribute *i*.

Final scores of the SBMS with different configurations are obtained. The candidate solution with the highest final score is determined as the best solution.

## 4. Case Study

### 4.1. Scenario Specification

A scenario environment from [26] was utilized in this case study. It was assumed that the SBMS will be installed in a rural district with about a 10-km distance between the transmitter and receiver. Appropriate large-scale fading will be applied. Besides, it was assumed that the battery was applied in the data center. Then, mobility is not necessary. Furthermore, the SBMS aimed to monitor the internal temperature, which required an adaptive report for the rapid monitoring. Hence, the low latency is demanded in this scenario.

### 4.2. Candidate Solutions

Three candidate solutions were proposed to illustrate the usage of SBMS-ES.

#### 4.2.1. Solution I

This section proposes an AI-based battery internal temperature monitoring approach for SBMS as a case study. Specifically, two features were measured using the sensors, i.e., ambient temperature and input current. Utilizing an AI algorithm, the internal temperature could be predicted with the MAE of about 5%. In addition, LoRaWAN was applied to implement a communication system with the spreading factor of 12 using Class A (i.e., an operation mode for LoRaWAN device). The EIRP was assumed to be 14 dBm (i.e., decibel-milliwatts), while the sensitivity of the receiver was −148 dBm [27].

First, the sensors were all installed outside the battery. Meanwhile, only two features were measured. Hence the sub-score was 5 for this aspect. The MAE was 5%. Hence, a sub-score of 3 was given for this attribute. LoRaWAN could meet the requirement of stationary applications. Hence, a sub-score of 3 was given for this attribute. The communication technology needed to satisfy the low latency requirement. Unfortunately, LoRaWAN cannot achieve low latency for the rapid monitoring. Hence, a sub-score of 2 was given for this attribute. As advised by [26], the fading was modeled using the Oulu model. After the fading calculation, the received power was estimated as −138 dBm, which is larger than the sensitivity of −148 dBm for LoRaWAN. Hence, a sub-score of 5 was given for this attribute.

#### 4.2.2. Solution II

This part proposed another SBMS solution. In this case, the temperature was directly measured using an internal sensor. The advantage of this method was the high accuracy, with the MAE of about 0.1%. Moreover, narrowband IoT (NB-IoT) was applied. It was assumed that the EIRP was 14 dBm, while the sensitivity was −135 dBm [27].

Similarly to the evaluation of Solution I, the scores for Solution II are given. The sub-scores for these five attributes are 2, 5, 3, 5, and 2.

#### 4.2.3. Solution III

The third solution was a combination of I and II. It utilized the AI-based prediction algorithm using two sensors installed outside the battery, with the MAE of 5%, as mentioned in Solution I. NB-IoT was utilized for communication, same as Solution II. The sub-scores for these five attributes are 5, 3, 3, 5, and 2 in Solution III.

### 4.3. Weighting Calculation

**Figure 2:**
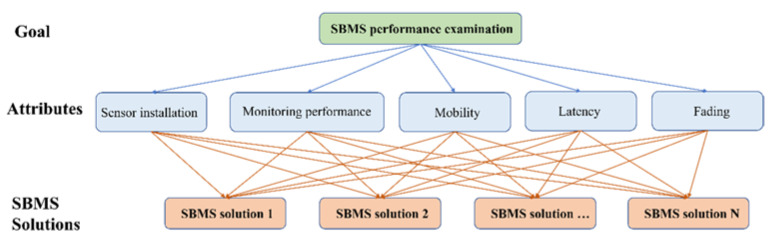
The structure of the AHP application for SBMS-ES.

Before calculating the final score, weighting allocation was implemented to decide the importance of the criteria in a specific scenario using AHP. The structure of the AHP application is shown in Figure 2. Regarding the goal of performance examination, the intensities of importance for the five attributes needed to be investigated. The degree of importance between every pair of attributes was obtained based on the scenarios’ requirements. The following shows a detailed example of weighting allocation using AHP.

Regarding this case, the temperature monitoring method for the batteries will be installed at the data center. Hence, the feature of mobility was not critically necessary. Thus, less weighting could be allocated to this attribute. The monitoring performance and sensor installation are important to ensure the battery system’s reliability. Hence, these two attributes required more weighting. Moreover, a priority level of latency and fading follows from this. As a result, a typical comparison matrix is provided as follows.

(7) $$C=\left[\begin{array}{ccccc} 1 & c_{12} & c_{13} & c_{14} & c_{15}\\ c_{21} & 1 & c_{23} & c_{24} & c_{25}\\ c_{31} & c_{32} & 1 & c_{34} & c_{35}\\ c_{41} & c_{42} & c_{43} & 1 & c_{45}\\ c_{51} & c_{52} & c_{53} & c_{54} & 1 \end{array}\right]$$

As *N* equals 5, *CR* could be written as:

(8) $$CR=\frac{\left(\lambda_{max}-5\right)/\left(5-1\right)}{1.12}$$

*CR* needs to be lower than 0.1 to pass the consistency check. Otherwise, the comparison matrix needs to be modified.

Utilizing Equations (3), (4), and (6), $w={\left[0.42,\;0.32,0.04,0.13,0.09\right]}^{T}$. After consistency checking using Equation (7), CR=0.04939<0.1. Hence, the weightings for the five attributes are 0.42, 0.32, 0.04, 0.13, and 0.09 respectively. The final score is calculated as 3.89. The final score indicates that the performance is good as a whole. However, it can be further improved.

### 4.4. Final Score Calculation

Based on the sub-scores and the weightings, the comprehensive performance of each SBMS solution could be evaluated, as shown in Table 4. Regarding Solution I, it did not perform well for the latency, but performed well on sensor installation and fading. As for Solution II, the inside-installed sensor decreased its sub-scores for sensor installation. However, this scheme also achieved high sub-scores for monitoring performance. As a result, the developer could alter the IoT technique from LoRaWAN to NB-IoT for Solution I, which resulted in Solution III. The final score of Solution III was calculated as 4.01, which is the highest among the three candidates. Hence, Solution III is considered the most desirable configuration in such a scenario.

## 5. Conclusions

The popularity of SBMS increases the development of relevant applications. However, SBMS designers have difficulties determining the most desired solution, as there is no evaluation method for SBMS. To address this issue, this paper presents an IEEE P2668 compatible evaluation strategy for SBMSs, namely SBMS-ES. The SBMS-ES identifies five essential attributes for SBMSs and defines grading principles for each attribute. Furthermore, a final score between 0 and 5 for the SBMS is obtained using a weighted calculation of the sub-scores, where the weighting is given using the AHP. With the quantitative evaluation scores, designers can compare the different solutions of SBMSs, regarding the scenario, and determine the best candidate. Moreover, the disadvantages of their SBMSs that may induce failure can be found when evaluating the attributes and making the related adjustments. For example, appropriate IoT technologies can be recommended regarding the demands of the application scenario of the SBMS. In particular, the SBMS-ES is compatible with IEEE P2668, a developing global IEEE standard for IoT maturity. The development of SBMS-ES represents a pioneering study of IEEE P2668 in SBMS. More evaluation strategies could be designed for other IoT-related scenarios by referring to this work.

## Figures and Tables

**Table 1:** Attribute scoring principle for SBMS-ES.

Score Level	Sensor Installation	Monitoring Performance	Mobility	Latency	Fading
1	Inside, large sensor no.	MAE > 10%	Mobile, not satisfied	High latency, not satisfied	Short distance, not satisfied
2	Inside, small sensor no.	10% ≥ MAE > 5%	Mobile or stationary, not satisfied	Low latency, not satisfied	Long distance, not satisfied
3	Outside, large sensor no.	5% ≥ MAE > 1%	Stationary, satisfied	High latency, satisfied	Short distance, satisfied
4	Outside, medium sensor no.	1% ≥ MAE > 0.5%	Mobile, satisfied	Medium latency, satisfied	Medium distance, satisfied
5	Outside, small sensor no.	0.5% ≥ MAE	Mobile and stationary, satisfied	Low latency, satisfied	Long distance, satisfied

**Table 2:** The intensity of importance and relative numerical value.

Intensity of Importance	Numerical Value
Equal importance	1
Moderate importance	3
Essential or strong importance	5
Very strong importance	7
Extreme importance	9
When a compromise is needed	2, 4, 6, 8

**Table 3:** The *RI* values for various *N*.

***N***	1	2	3	4	5	6	7	…
***RI***	0	0	0.58	0.90	1.12	1.24	1.32	…

**Table 4:** Case feature description and evaluation.

Item	SBMS-ES Score					
	Sensor Installation	Monitoring Performance	Mobility	Latency	Fading	Final Score
Solution I	5	3	3	2	5	3.89
Solution II	2	5	3	5	2	3.39
Solution III	5	3	3	5	2	4.01
Weighting	0.42	0.32	0.04	0.13	0.09	1

## Data Availability

Not applicable.
